# Valuing the evidence on manual therapy in radius fractures: comments on the study by Horoz and colleagues

**DOI:** 10.1590/1806-9282.20250928

**Published:** 2025-12-05

**Authors:** Camila Vereau Estrada, Camila Moscol Zarria

**Affiliations:** 1 Universidad Privada San Juan Bautista, Facultad de Ciencias de la Salud, Especialidad en Terapia Física y Rehabilitación – Lima, Perú.

Dear Editor,

We read with interest your recent article titled "Effectiveness of mobilization with movement in patients operated for distal radius fracture (DRF): a single-blinded, randomized controlled study." Our aim is to highlight that the individualization of the Mulligan technique [Mobilization with Movement (MWM)] should be based on each patient's characteristics to optimize its effectiveness in postoperative rehabilitation. In this regard, we would like to offer some reflections on your findings related to the MWM technique, emphasizing that a proper application of its principles is essential to achieve better therapeutic outcomes, especially during the early phases of rehabilitation after a DRF.

DRFs are among the most common orthopedic injuries. In the United States, there are about 67 upper limb fractures per 10,000 people per year, approximately one-fourth of which involve the distal radius and ulna. The age distribution is bimodal: in children and adolescents, these fractures are usually due to falls during sports or play, whereas in older adults—especially postmenopausal women—they are associated with low-impact falls in the context of osteoporosis. The most common injury mechanism is a fall on an outstretched hand, although in the elderly, factors such as chronic corticosteroid use, cognitive disorders (e.g., dementia), and a history of fragility fractures also contribute. Poor management of these fractures can lead to significant functional complications, negatively impacting the patient's quality of life^1^.

Much of the physiotherapeutic intervention is based on Orthopedic Manual Therapy, which has evolved significantly in recent decades by incorporating various techniques aimed at optimizing patients’ functional recovery. Among these, the Mulligan concept, which introduces the MWM technique, has gained recognition for its innovative approach and promising results in treating various musculoskeletal conditions. In the context of DRFs—one of the most common upper limb injuries—the use of MWM has been explored as a therapeutic strategy to improve range of motion, reduce pain, and accelerate functional recovery.

Your study makes a valuable contribution by rigorously evaluating the effects of the Mulligan technique (MWM) in patients with surgically treated DRFs. Your results show significant but selective improvements, particularly in grip strength, where the intervention group reached 21.5±5.2 versus 15.8±3.5 kg in the control group (p<0.001), and in the range of pronation, with 83.9°±6.2 versus 84.3°±7.7 (p<0.05)^2^. These findings contrast notably with those reported by the study "Effects of early manual therapy on functional outcomes after volar plating of DRFs: A randomized controlled trial," where earlier application of MWM (initiated between 7 and 10 days post-surgery) showed broader benefits, including greater improvement in wrist flexion (71.0°±12.2 vs. 54.4°±15.5), greater grip strength (24.05 vs. 16.12 kg), and a marked reduction in pain [visual analog scale (VAS 0.58 vs. 3.3)]^3^.

This divergence in results invites reflection on fundamental methodological aspects that could explain the observed differences, such as the timing of intervention initiation, the individualized treatment approach, and the specific application of MWM techniques. The timing of the intervention emerges as a critical factor, as your study began MWM 2 weeks post-surgery, whereas the study by Tomruk et al.^3^ started significantly earlier (7–10 days). This temporal detail is particularly relevant, as Tomruk et al. demonstrated that early manual intervention after volar fixation of DRFs reduces joint stiffness and significantly improves functional recovery in the early weeks of rehabilitation. Moreover, the individualized treatment approach aims to identify the criteria guiding expert physiotherapists in selecting and applying MWM, considering factors such as patient assessment, prediction of treatment responses, most receptive body areas, and expected outcomes. The selection and application of MWM require an individualized approach: although your study used a standardized protocol, Mulligan's original principles emphasize the need to adjust the direction of the glide to correct the patient's specific positional faults during active, pain-free movement^4^. According to Mulligan, mobilizations with movement must meet three essential criteria: they must not cause pain during application, produce immediate improvement in the range of motion, and be performed in repetitions with progressive overload^5^. These principles could also explain why some studies report broader and more consistent MWM effects, while others find benefits limited to certain functional aspects. It would be of great interest to know to what extent these criteria were met during your intervention, as strict adherence could significantly influence the outcomes obtained.

Your study represents a valuable contribution to the clinical knowledge of applying the Mulligan technique (MWM) in patients operated on for DRFs. However, the findings encourage deeper consideration of critical variables such as the timing of intervention initiation, the need to individualize treatment, and the rigorous application of the method's fundamental principles. Comparative evidence suggests that an early and tailored implementation of MWM could enhance its functional benefits, including greater improvement in the range of motion (especially wrist flexion and pronation), a significant increase in grip strength, and marked pain reduction. In this regard, we suggest that future research incorporate personalized clinical criteria and evaluate different therapeutic windows to optimize outcomes in postoperative rehabilitation of these fractures.

## Data Availability

The datasets generated and/or analyzed during the current study are available from the corresponding author upon reasonable request.

## References

[1] Corsino CB, Reeves RA, Sieg RN (2025). Distal radius fractures.

[2] Horoz L, Cigdem-Karacay B, Ceylan I, Alkan H (2024). Effectiveness of mobilization with movement in patients operated for distal radius fracture: a single-blinded, randomized controlled study. Rev Assoc Med Bras (1992).

[3] Tomruk M, Gelecek N, Basçi O, Özkan MH (2020). Effects of early manual therapy on functional outcomes after volar plating of distal radius fractures: a randomized controlled trial. Hand Surg Rehabil.

[4] Baeske R, Silva MF, Hall T (2020;). The clinical decision making process in the use of mobilisation with movement - A Delphi survey. Musculoskelet Sci Pract.

[5] Gutiérrez-Espinoza H, Araya-Quintanilla F, Olguín-Huerta C, Valenzuela-Fuenzalida J, Gutiérrez-Monclus R, Moncada-Ramírez V (2022). Effectiveness of manual therapy in patients with distal radius fracture: a systematic review and meta-analysis. J Man Manip Ther.

